# Cochlear implantation in syndromic patients: difficulties and lessons learnt

**DOI:** 10.1007/s00405-024-08897-2

**Published:** 2024-08-29

**Authors:** Mina Fayez Saleeb, Lobna El Fiky, Badr Eldin Mostafa, Ossama Mustafa Mady, Ahmed Abdelmoneim Teaima

**Affiliations:** Otorhinolaryngology Department Faculty of Medicine, Ain Shams University, Ramses Street, Abasseyia Square, Cairo, 11566 Egypt

**Keywords:** Syndromic hearing loss, Cochlear implantation, Inner ear anomalies, Congenital hearing loss

## Abstract

**Objective:**

Identify the prevalence of syndromes in a cohort of patients who underwent cochlear implantation, to report on the presence of inner and middle ear malformations and highlight the surgical difficulties encountered.

**Study design:**

Observational, retrospective study.

**Setting:**

Tertiary referral children’s hospital pediatric cochlear implant program.

**Material & methods:**

An IRB-approved retrospective chart review of children undergoing cochlear implantation at a tertiary academic medical center, from 2018 to 2023. Preoperative imaging data of syndromic patients in that cohort with special attention to the presence of inner ear or middle ear malformations were collected. Abnormal intraoperative findings and difficulties reported by the surgeons were also noted.

**Results:**

1024 children were unilaterally implanted for bilateral profound hearing loss. There were 45 cases diagnosed with associated syndromes (4.3%). The commonest syndromes were Jervell and Lange Nielsen (JLN) syndrome followed by Waardenberg syndrome (WS), in a prevalence of 34% and 32% respectively. These syndromes had no associated inner ear malformations (IEM). Less common syndromes included Branchio-oto-renal (BOR) syndrome, CHARGE association and Treacher Collins syndrome, 3 cases each, and keratosis icthyosis deafness syndrome (KID), Usher syndrome and Albino, 2 cases each and an H syndrome case. There were 9 cases (20%) with IEM, with 6 cases of perilymph gusher. Two cases had middle ear anomalies and one case had a facial nerve course abnormality. The outcome of these cases was similar to non-syndromic cases.

**Conclusion:**

Children with syndromic HL should be dealt with on a case by case scenario to diagnose inner and middle ear malformations. Additional disabilities can affect the rehabilitation procedures. All children with congenital hearing loss should undergo pediatric, cardiologic, ophthalmologic and nephrologic examination in order to exclude the syndromic etiology of hearing loss.

## Introduction

Syndromic children with congenital profound sensorineural hearing loss (SNHL) are a unique population, having one or more associated co-morbidities, that may impact the cochlear implantation (CI) or the rehabilitation procedures [1]. There is a large number of syndromes related to congenital hearing loss (HL). Most of these cases can be diagnosed by visible traits and co-morbidities. However, some syndromes are not apparent and may pass undiagnosed or delayed.

It is important to be familiar with the wide range of syndromes involving HL for a proper early diagnosis. Early intervention can be pursued to establish, preserve, or restore functional hearing [2]. It also facilitates counseling and is paramount to achieve safe and effective outcomes in this special population [1].

Different syndromes can be associated with inner ear malformations making CI surgery challenging. About 30-40% of patients with congenital HL have other disabilities as visual and learning disorders [1, 2]. Outcome is often excellent but can be variable even within the same syndrome group. Such children are therefore assessed on an individual basis to ensure a realistic expectation [2].

In this retrospective case series in a tertiary referral cochlear implant center, we describe a series of children with HL due to a clinical syndrome who underwent CI. We conducted this study to identify the prevalence of syndromic HL in this cohort as well as the commonest syndromes encountered. The incidence of inner and middle ear anomalies as well as difficulties during surgery were also noted.

## Materials and methods

An IRB-approved (FMASU R359/2023) retrospective chart review of children undergoing CI at a tertiary academic medical center, Cairo, Egypt, in the period from January 2018 to January 2023. Demographic data, radiology and operative findings for syndromic patients were collected. The presence of inner ear, middle ear malformations or facial nerve anomalies were looked for. Abnormal intraoperative findings and difficulties reported by the surgeons were also noted.

## Results

This cohort included 1024 children who underwent unilateral CI for bilateral profound HL. There were 45 cases diagnosed with associated syndromes (4.3%), 17 of them were males and 27 females (Table 1). All cases were prelingual children less than 5 years, except for one post-lingual child with H syndrome (10 years old) and 2 Albino children (8 & 12 years old). There was positive consanguinity in 36% of cases of first cousins’ degree paternal & maternal equally.

**Table 1:** Demographic data of patients

Syndrome	Number	Age (mean age)	Sex	
			Male	Female
JLN	15	3.5	6	9
Waardenburg	14	2.9	5	9
Branchio-Oto-Renal	3	4	2	1
Keratosis ecthyosis deafness	2	5	1	1
Albino with Profound SNHL	2	3.2	1	1
CHARGE	3	4.2	2	1
Treacher Collins	3	4	0	3
Usher	2	4.7	0	2
H syndrome	1	5.5	1	0

**Fig. 1:**
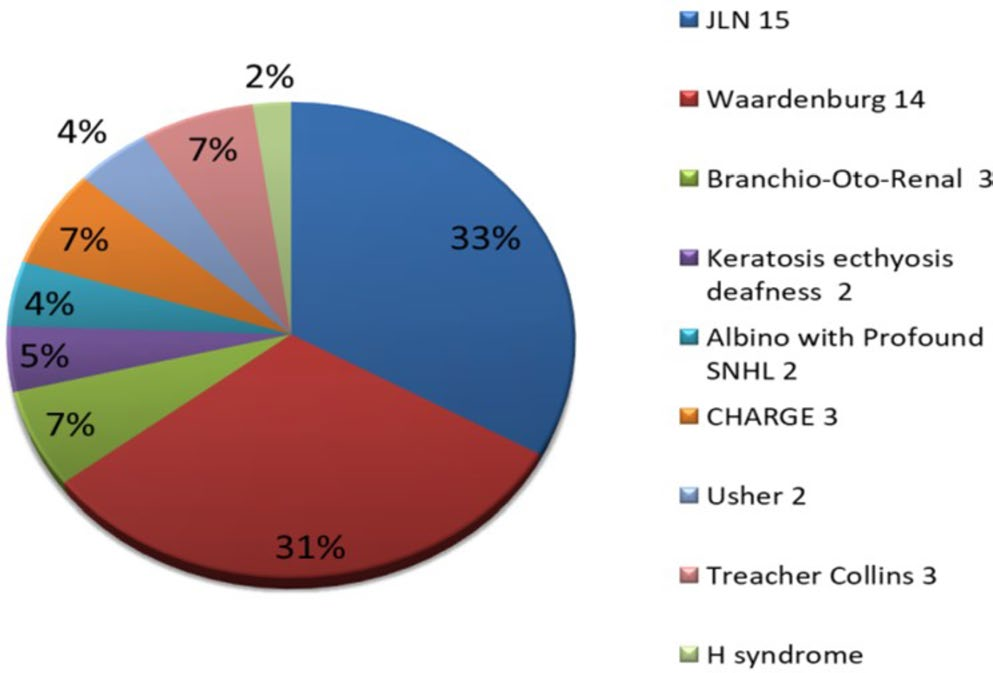
Syndromes in our study

Nine syndromes could be diagnosed (Fig. 1). The commonest were Jervell and Lange-Nielsen syndrome (JLN) and Waardenburg syndrome (WS) with a prevalence of 33% and 31% respectively.

### Jervell and lange nielsen syndrome (JLN)

There were 15 patients with this syndrome with a mean age of 2.9 years. Only one case was diagnosed before implantation and was on medical treatment. The rest were diagnosed by the anesthesiologist reviewing the routine preoperative ECG done for these patients which showed long QT interval (LQTc = 500–635 msec). One child developed severe bradycardia during induction and was reverted by cardiac massage. Nine JLNS cases were wrongly diagnosed as petit mal epilepsy and were on anticonvulsant treatment due to history of repeated attacks of near fainting. One of the children with JLN had undiagnosed sibling who suddenly died. All cases were on beta blockers before surgery, were closely monitored during the surgery to avoid tachycardia and received magnesium sulfate during operation. They were post-operatively referred to a pediatric cardiologist for further management.

### Waardenburg syndrome (WS)

This was the second most common syndrome. There were 14 cases [5 cases WS 1 and 9 cases WS 2]. They were easily diagnosed due to characteristic phenotypic features of hypopigmentation, white forelock of the hair, heterochromia iridis and white lashes. WS2 only differs in the absence of epicanthus. They all had first degree relative affection.

All cases of JLNS and WS had no middle or inner ear anomalies on imaging and implantation was straightforward.

### Less common syndromes (*n* = 16)

In 7 cases, there were no inner ear anomalies with uneventful surgery.

**Fig. 2:**
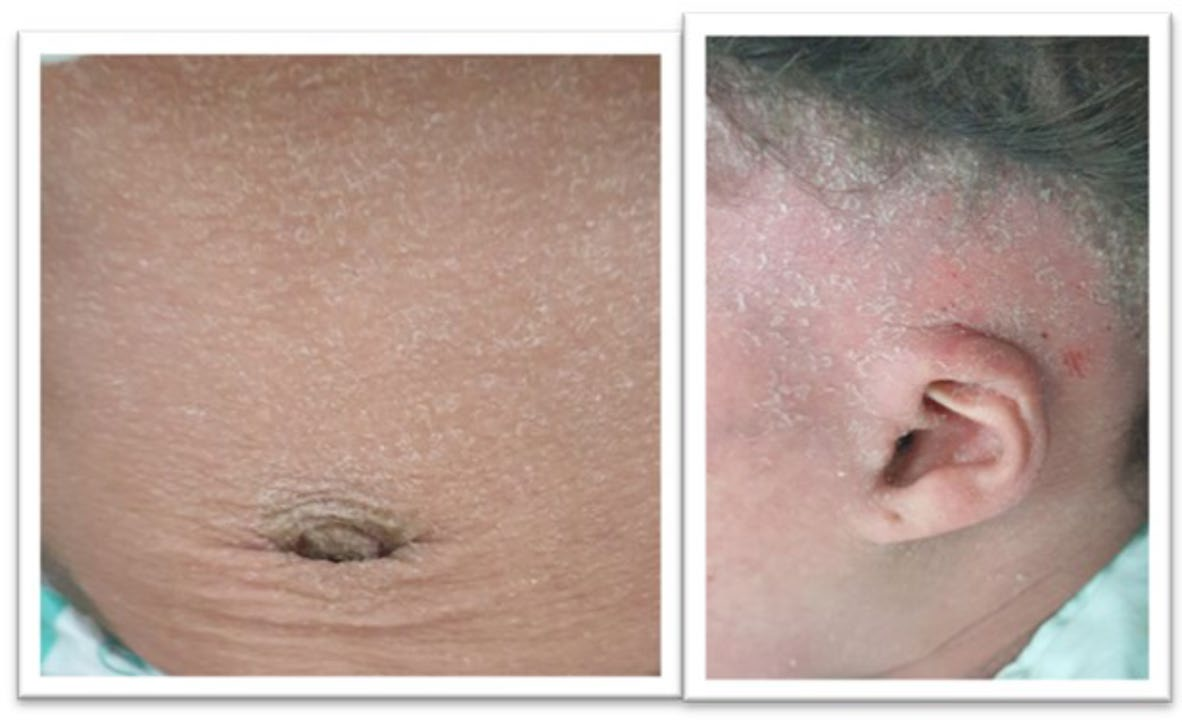
KID syndrome features

- Keratitis Icthyosis deafness syndrome (KID): These were 2 female siblings, both on regular local skin care for the disease. Prophylactic antibiotics were extended post-operatively until complete healing, without any skin dehiscence (Fig. 2).
- Usher syndrome (*n* = 2), one case was diagnosed by the presence of retinitis pigmentosa by electroretinography causing tubular vision and the other had nystagmus and vestibular symptoms.
- Albino (*n* = 2): The characteristic hypopigmentation of skin, hair and eyes is easily spotted. These were both post-lingual cases.
- H syndrome (*n* = 1): This was a 10 year-old post-lingual child with hyperpigmented skin lesions and hypertrichosis, hepatosplenomegaly, hypogonadism, and cervical lymphadenopathy.

In the remaining 9 cases (20%) there were inner ear anomalies, with a total of 6 cases of perilymph gusher.

**Fig. 3:**
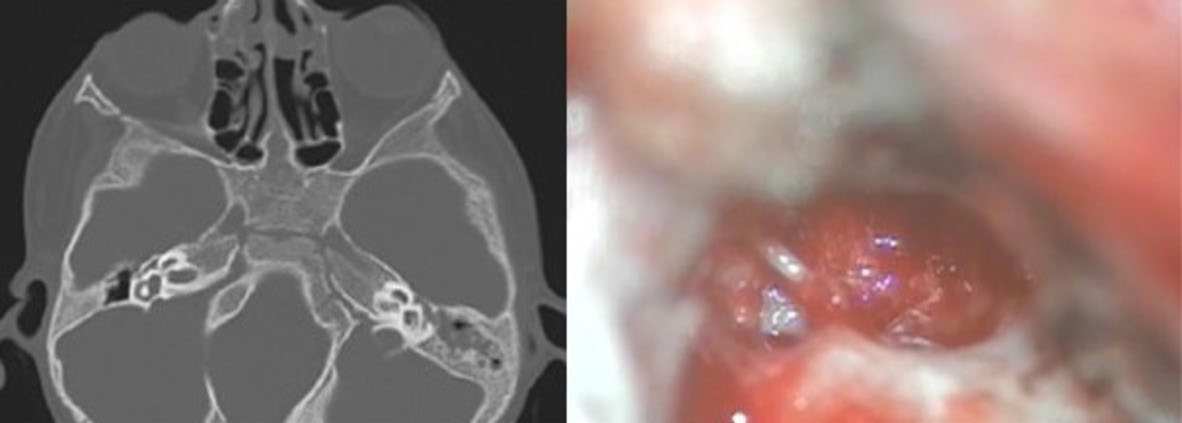
Isolated WVA & Ossicular anomalies in Treacher-Collins case

- Branchio-oto-renal syndrome (BOR: *n* = 3) were characterized by cupped auricle, skin tags, cervical sinus. 2 cases had IP2 inner ear anomaly and the last had isolated wide vestibular aqueduct.
- CHARGE association (*n* = 3): One extreme case had karyotyping which showed 46,XY.ish 22q11.2(LSI N25 × 2) mutation. Multiple comorbidities were present as choanal atresia, microphthalmia, Dandy-Walker anomaly, Tetralogy of Fallot’s as well as Esophageal atresia. He underwent canalization of choanae, heart surgery as well as fundoplication. Inner ears showed unilateral cochlear hypoplasia type 2 with cochlear nerve deficiency and contralateral IP2 with wide vestibular aqueduct. The latter side was implanted and helped in improving the quality of life of this patient.
- Treacher-Collins syndrome (TCS: *n* = 3): 2 cases had IP2 anomaly and one case isolated wide vestibular aqueduct (Fig. 3).

Middle ear anomalies were found in 2 cases. Ossicular anomalies were present in a case of TCS with an abnormally situated round window niche (Fig. 3). There was also an infantile antrum with anteriorly displaced sigmoid sinus necessitating subtotal petrosectomy. An abnormal facial nerve passing over the round window was encountered in a case of CHARGE syndrome. After parents counselling, the nerve was gently manipulated to perform the cochleostomy, resulting in postoperative partial facial nerve paresis, that improved in 4 months.

**Fig. 4:**
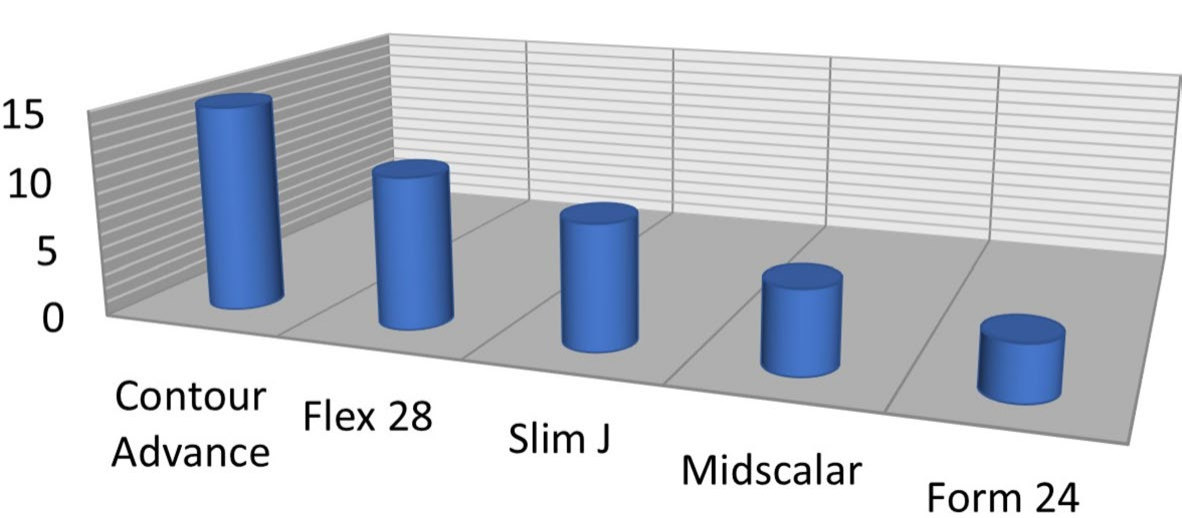
Used electrodes

Different electrodes were used: Cochlear^®^ contour advance in 15 cases, Advanced Bionics^®^ midscalar in 6 cases and Slim J in 9 cases, Med-El^®^ Flex 28 in 11 cases and Form 24 in 4 cases. Full electrode insertion was achieved in all cases (Fig. 4).

Postoperative audiological results were satisfactory and comparable to a similar cohort of non-syndromic children. Pure tone audiometry ranged from 25 to 40dB with mean score of 30dB. Word discrimination score ranged from 28 to 96% with a mean score of 63% (see Table 2).

**Table 2:** Syndromes details

Syndrome	*N*	M.E. anomalies	Inner E. anomalies	Intraoperative difficulties	PTA (mean)	WDT (mean)
JLN	15			One case intraoperative arrythmia.	31	76
Waardenburg	14				30	78
Branchio-Oto-Renal	3		IP2, WVA	One case CSF gusher	28	70
Keratosis ecthyosis deafness	2				30	85
Albino with Profound SNHL	2				25	80
CHARGE	3	One case: Abnormal facial n. over RW.	WVA, cochlear hypoplasia and chochlear n. hypoplasia, IP2	facial transposition	32	81
Treacher Collins	3	One case: ossicular anomalies	IP2, WVA	One case CSF gusher	30	64
Usher	2				40	52
H syndrome	1				32	68

## Discussion

A diverse heterogenous group of abnormalities are associated with deafness in syndromic HL. These comorbidities can be visual, skeletal, endocrinal, neurological, skeletal, cardiac, renal, craniofacial, metabolic anomalies [3–5]. These disabilities, as visual and learning disorders, could seriously affect the rehabilitation process outcome. However, cochlear implantation improves quality of life of such a group of children and help their care givers to communicate with them by increasing environmental sound awareness [6].

In a study conducted by Broomfeld et al. on syndromic patients, they found a variation in the outcome within the children of the same syndrome. They recommended assessment of these cases on individual basis for CI [2]. There is usually a difference in the penetrance of comorbidities among patients necessitating individual consideration of the handicap of each child. A clear example is the case of extreme CHARGE with multiple severe anomalies, making the implantation the only possible means of communication. In the study conducted by Kay-Rivest et al., they found satisfactory audiological results, and they recommended consideration of CI in CHARGE syndrome to maximize auditory and sensory inputs [7].

The incidence of different syndromes in different populations is not clearly addressed in the literature. The commonest syndromes we encountered in this cohort were found to be JLN (34%) and WS (32%) respectively. This is the first study reviewing syndromic cases who underwent CI in the Egyptian population.

WS is very easily diagnosed by the characteristic phenotypic appearance as well as positive family history. In contrast, JLN usually passes undiagnosed as seen in our study. This implies the importance of doing an ECG routinely for all children undergoing CI, to spot these cases pre-operatively and start medical treatment which can dramatically decrease the incidence of cardiac events. Once diagnosed, this will allow performing the surgery safely. A whole protocol is followed by the anesthesiologist to avoid use of dangerous drugs, tachycardia, hypotension, hypothermia, and electrolytes imbalance (Victoria Scott-Warren). The theater should also be ready to deal with any arrythmias that can develop. Kang et al. confirmed the importance of ECG as first sign of this serious syndrome [8]. In spite of diagnosis and medication intake in these children, follow-up with a cardiologist is essential, as they may need defibrillation or a pacemaker after implantation. JLNS is associated with high risk of sudden death in 25% cases. 50% of JLNS patients had cardiac event at 3 years old [8–10]. Broomfield et al. had two cases of JLN who suffered cardiac arrest then sudden death in spite of medical management [11]. Kaneshiro et al. reported JLN case needed defibrillation postoperative after CI surgery [12].

There were no inner ear malformations in any of the cases of JLN and WS, making the surgery straightforward. In the literature, WS can be associated with vestibular aqueduct and semicircular canals malformations [13, 14]. WS outcome of our patients was similar to non-syndromic cases in accordance with De Sousa Andrade et al. [15]. Yanmei et al. found that the hearing and speech outcomes in JLN patients after CI is like other non-syndromic cases [10].

Although the outcome of CI in these cases is not a part of our objectives in this study, but in conjunction with Caragli et al. 2023, the severity of cognitive impairment is inversely proportional to the degree of improvement in auditory and language skills. As these syndromes do not imply any cognitive impairment, the outcome is similar to non-syndromic cases.

The rest of the syndromes were much less common. Some were accompanied by IEM with high incidence of perilymph gusher during surgery, necessitating use of a corking electrode such as Form 24 of Med-EL^®^. Meticulous radiologic evaluation of these cases to spot the presence of IEM and the status of the cochlear nerve for any deficiencies is of paramount importance.

Middle ear anomalies can be found in syndromes with facial bones affection such as TCS, making the surgery even more difficult or necessitating surgical deviation from standard technique to a more drastic approach such as subtotal petrosectomy. Facial nerve course should be evaluated for any aberrations in cases such as CHARGE association, to avoid intraoperative injury. This will allow counselling of the patient as well as choice of the side to be implanted.

Some syndromes such as KID, necessitate careful surgical incision planning to avoid wound dehiscence due to the inherent skin defect. Prolonged follow-up and explaining the importance of the long-term skin flap care for the parents is essential to avoid skin dehiscence over the device.

Visual co-morbidities present in some syndromes such as retinitis pigmentosa in Usher syndrome, and in CHARGE association and for Albino cases, are considered a big challenge for rehabilitation of such cases. Loundon et al., enhanced the need for early detection of these cases by electroretinography in any child with congenital HL [16]. These cases necessitate early implantation before total visual loss, and preferably bilateral in such a population. Unfortunately, only one device is reimbursed for each child, but we need to implement such a regulation for this group of patients.

CI proved more in these cases to be a multidisciplinary process, as multiple subspecialities may be involved in the decision making and care of these patients, such as genecists, cardiologists, ophthalmologists, anesthesiologists and others. Our study lacked the genetic testing of such cases as it is not routinely available.

## Conclusions

CI is a successful rehabilitative treatment for syndromic HL. Audiological and speech outcomes are satisfactory. Cases should be studied well before surgery on individual basis and surgeons should be ready for challenges intraoperatively and postoperatively. Counselling of parents should be done according to the expectations of associated co-morbidities. All children with congenital hearing loss should undergo pediatric, cardiologic, ophthalmologic and nephrologic examination in order to exclude the syndromic etiology of hearing loss.

## Data Availability

Data is available on request.
